# Down-regulation of HSP70 sensitizes gastric epithelial cells to apoptosis and growth retardation triggered by *H. pylori*

**DOI:** 10.1186/1471-230X-11-146

**Published:** 2011-12-30

**Authors:** Weili Liu, Yan Chen, Gaofeng Lu, Leimin Sun, Jianmin Si

**Affiliations:** 1 Gastroenterology laboratory, Clinical Research Institute, Sir Run Run Shaw Hospital, School of Medicine, Zhejiang University, 310016 Hangzhou, People's Republic of China; 2 Department of Gastroenterology, Second Affiliated Hospital, School of Medicine, Zhejiang University, Hangzhou, People's Republic of China; 3 Department of thoracic surgery, Zhejiang Hospital, Hangzhou, People's Republic of China

## Abstract

**Background:**

*H. pylori *infection significantly attenuated the expression of HSP70 in gastric mucosal cells. However, the role of HSP70 cancellation in *H. pylori*-associated cell damages is largely unclear.

**Methods:**

Small interfering RNA (siRNA) was used to down-regulate HSP70 in gastric epithelial cell lines AGS. The transfected cells were then incubated with *H. pylori *and the functions of HSP70 suppression were observed by viability assay, cell cycle analyses and TUNEL assay. HSP70 target apoptotic proteins were further identified by Western blot.

**Results:**

The inhibition of HSP70 has further increased the effect of growth arrest and apoptosis activation triggered by *H. pylori *in gastric epithelial cells. The anti-proliferation function of HSP70 depletion was at least by up-regulating p21 and cell cycle modulation with S-phase accumulation. An increase of apoptosis-inducing factor (AIF) and cytosolic cytochrome C contributes to the activation of apoptosis following down-regulation of intracellular HSP70. Extracellular HSP70 increased cellular resistance to apoptosis by suppression the release of AIF and cytochrome c from mitochondria, as well as inhibition of p21 expression.

**Conclusions:**

The inhibition of HSP70 aggravated gastric cellular damages induced by *H. pylori*. Induction of HSP70 could be a potential therapeutic target for protection gastric mucosa from *H. pylori*-associated injury.

## Background

In recent years, heat shock proteins (HSP) have been implicated to be an additional factor utilized for the gastric defence mechanisms at the intracellular level [1]. HSP70 is generally considered to be a major molecular chaperone to accelerate the cellular recovery from different stimuli by cope with unfolded or denatured proteins [2], through which HSP70 might achieve efficient mucosal defence for ulcer or inflammation healing [3,4].

Helicobacter pylori (*H. pylori*) infection leads to significant inflammations in the gastric mucosa, which is closely associated with development of atrophic gastritis, peptic ulcer, gastric cancer, and mucosa-associated lymphoid tissue (MALT) lymphoma. Animal studies have demonstrated that *H. pylori *infection damages gastric mucosa by either disrupting the balance in cell apoptosis and proliferation, or decreasing migration of epithelial cells within the gastric mucosa [1,5,6]. Recent studies have found that *H. pylori *decreases the synthesis of HSP70 in gastric epithelial cells by the inactivation of heat shock factor- 1 [7-11], however, whether the inhibition of HSP70 would be the prominent event leading to the persistent damages from *H. pylori *in gastric epithelial cells remains unclear.

*H. pylori *produces ammonia in gastric mucosa with its high urease activity. Our previous animal studies have introduced ammonia solution to simulate the conditions of *H. pylori *infection, and succeeded in inducing atrophic gastritis in rats [12]. Further studies demonstrated that induction of HSP70 expression is beneficial for preventing gastric atrophy and maintaining mucosal functions in gastric cells [12]. Since the induction of HSP70 is suggested to constitute a novel therapeutic approach for the prevention or treatment of *H. pylori*-associated conditions, it's conceivable that deregulation of HSP70 might be a prominent cause of *H. pylori*-associated damages. Therefore, we investigated the correlation of HSP70 inhibition with the mucosal damages induced by *H. pylori *in this study.

## Methods

### Cell culture and transfection

Human gastric epithelial cell line AGS (CRL-1739, ATCC, USA) were maintained in RPMI1640 medium supplemented with 10% fetal bovine serum (FBS) without antibiotics at 37°C in a humidified atmosphere of 5% CO_2 _and 95% air.

Small interfering RNAs (siRNAs) were designed against the mRNA sequences targeting HSP70 (Genebank: NM_005345.5), siRNA_1_: 5'-CTTTCCAGGTGATCAACGA-3', siRNA_2_: 5'-AGGACGAGTTTGAGCACAA-3'[13], siRNA_3_: 5'-GACTTTGCATTTCCTAGTA-3'. We used RNAi-Ready vector, which contains a neomycin resistance gene and GFP for selection of stable transfectants. In the preliminary experiments, we employed three constructs that target three distinct regions of the HSP70 gene to deplete HSP70 expression, and found siRNA_2 _was better than the others for the short-term inhibition of HSP70. Therefore, siRNA_2 _was selected for the following stable transfection. AGS cells were transfected with the HSP70 siRNA constructs by use of lipofectamine according to the manufacturer's protocol. The total amount of plasmids was adjusted by using the empty vector plasmid in each assay. Briefly, 1 ×10^5 ^cells were plated in RPMI1640 containing 10% FBS in 6-well plates 24 h before transfection. Then transfection was performed with serum-free RPMI1640 containing 2 μg plasmid constructs and 6 μl lipofectamine. After 5 h, fresh RPMI1640 containing 10% FBS was added until 2 ml of final volume. The selection with 0.4 mg/ml neomycin was started 48 h after transfection. GFP was used as a control for transfection or selection efficiency. A control sample transfected with empty vector plasmid was included. Neomycin-resistant cell pools and single cell clone were generated, in which HSP70 expression was confirmed by immunoblot analysis and real-time PCR.

### Bacterial strain and coculture conditions

*H. pylori *expressing CagA and VacA (ATCC 700392) were grown on Columbia agar medium with 5% of fresh sheep blood under microaerobic conditions (5%O_2_, 10%CO_2_, 8%N_2_) at 37°C. Before the experiment, bacteria were harvested and suspended in RPMI 1640 medium (including 10% FBS but no antimicrobial agents). The bacteria were densitometrically counted according to the McFarland scale and suitable dilution was prepared for the cell culture (bacteria/cell ratio at 200:1 for most tests).

### Real-time PCR

The RNA was harvested from cell culture with RNeasy columns (QIAGEN). Single stranded cDNA synthesis was made with the TaqMan RT Kit (QIAGEN) using oligo-(dT)_16 _primers. The cDNA originating from the transfected cells were used as template for the following PCR reaction and the housekeeping gene glyceraldehyde-3-phosphate dehydrogenase (GAPDH) was served as an internal control. Primers were designed as follows (Genebank: NM_005345.5): HSP70-for, 5'-AACACCGTGTTTGACGCGAA-3'; HSP70-rev, 5'-GGTCAGCACCATGGACGAGA-3'; HSP70-probe, 5'-FAM-CCAGGTGATCAACGACGGAGACAAGCCC-TAMRA-3'. The negative control contained the reaction mixture but no DNA. The reactions were performed with a real-time PCR machine (BIOER, Japan) with a Taq activation at 95°C for 5 min followed by 35 cycles of three segments consisting of 30 sec at 95°C, 30 sec at 55°C, and 30 sec at 72°C. The level of HSP70 mRNA was evaluated relative to that of GAPDH mRNA.

### Growth curve and cell proliferation Assay

Cell growth curve or proliferation assessment was quantified using a tetrazolium salt colorimetric assay with 3-[4,5-dimethylthiazol-2-yl]-2,5-diphenyltetrazolium bromide (MTT, final concentration of 0.5 mg/ml, Sigma-Aldrich, St. Louis, MO, USA). Briefly, the cells stably transfected with HSP70 siRNA or the empty vector were cultured in a 96-well plate for 1~6 days. In the proliferation assay, these cells were incubated with live *H. pylori *for 0, 24, 48 or 72 hours. The absorbance of samples was measured at 492 nm in the microplate reader.

### Cell apoptosis assays

Cells suspension (2 × 10^4^) was added to each well of 48-well plates and was incubated with *H. pylori *(1:200) for 24 h. We analyzed apoptosis with the use of the terminal deoyecelotibyl transferase mediated dUTP-biotin nick end labeling assay (TUNEL) kit (Cell Death Detection kit, Roche, Germany) according to the instructions provided by the manufacturer. Quantitation of apoptotic cells was accomplished by counting the number of apoptotic bodies sighted in the microscopic fields. Labeling indices were calculated as the mean number of labeled cells (from five random fields of vision) divided by total counted cells (500 cells).

### Cell cycle analysis

Cells were seeded (2 × 10^5^) on 6-well plates and were synchronized through serum starvation for 48 hours. Then the cells were incubated with *H. pylori *at 200:1 of bacterium to cell ratio in RPMI1640 containing 10% FBS for 0, 6, 12 or 24 hours. The treated cells were collected and fixed in 70% ethanol. Cell pellets were resuspended in 500 μl of propidium iodide buffer (10 mM Tris-Cl at PH 7.5, 50 μg/ml propidium iodide, 0.1% Triton X-100, 0.1% sodium citrate and 2 mg/ml RNase) and incubated in the dark at 4°C overnight. Stained cells were analyzed by the Beckman Coulter EPICS XL flow cytometer using the CellQuest software. At least 1 × 10^4 ^cells have been tested in each test.

### Immunoblotting analyses

Total proteins were isolated from the transfected cells and the concentration was measured by Bio-Rad Protein Assay. Mitochondrial or cytosolic protein was extracted in according to the protocol of Mitochondria/cytosol Fractionation Kit (BioVision). All procedures were performed at 4°C. The expressions of HSP70 (anti-HSP70, Sigma-Aldrich, St. Louis, MO, USA), Bax, caspase-3, caspase-6, caspase-7, cytochrome c, p21, PCNA (antibodies, Cell Signaling Technology, Danvers, MA, USA) and AIF (anti-AIF, Santa Cruz Biotechnology, Santa Cruz, CA) were assessed by immunoblotting with corresponding antibodies. The blotted membrane was visualized by chemiluminescent substrate (EZ-ECL, Kibbutz Beit Haemek Israel). The immunoblotting for β-actin (Santa Cruz Biotechnology, Santa Cruz, CA) was used as a loading control.

### Statistical analysis

Any significance in differences between two data sets was determined by the Student's *t-*test. *P *values < 0.05 were considered significant in all analyses.

## Results

### *H. pylori *infection suppressed HSP70 expression in gastric cell line AGS cells

**Figure 1:**
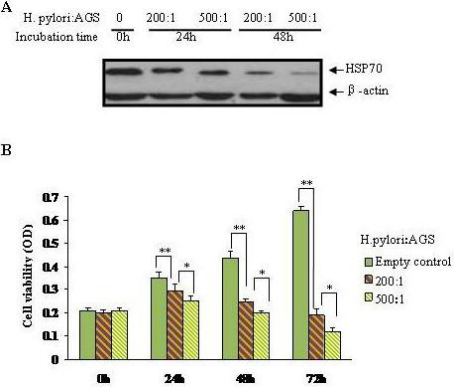
**Effects of *H. pylori *on AGS cells**. (A) AGS cells were treated with *H. pylori *at the ratio of 200:1(bacterium to cells) or 500:1 for 0, 24 or 48 hours, respectively. Suppression of HSP70 in AGS by *H. pylori *infection was evidenced by immunoblotting. (B) *H. pylori *infection significantly inhibited cell proliferation in gastric epithelial cell line in a dose- and time-dependent manner. Data are mean ± SD, *P <0.05, **P <0.01.

We first examined the influence of *H. pylori *on HSP70 expression in AGS cells. Basal HSP70 expression was found relatively high in the gastric epithelial cells, but *H. pylori *infection induced a significant suppression of HSP70. The inhibitory effect of *H. pylori *on HSP70 expression was improved with the extended incubation time from 0 h up to 48 h. The signal for HSP70 protein was detectable in AGS cells incubated in the medium containing *H. pylori *at 200:1 or 500:1 of bacterium to cell ratio, and the suppression of HSP70 in response to bacterium appeared to be concentration-dependent manner (Figure 1. A). We also explore the effect of the live bacteria on the viability of AGS cells over different periods of time. Concomitant with HSP70 inhibition by *H. pylori*, incubation of *H. pylori *with AGS cell line caused a significant decrease in cell viability. Coincidentally, the inhibitory effect of *H. pylori *on AGS cell growth was improved with the increased concentration of the bacterium and extended incubation period (Figure 1. B).

### Generation of gastric cells AGS with reduced level of HSP70

**Figure 2:**
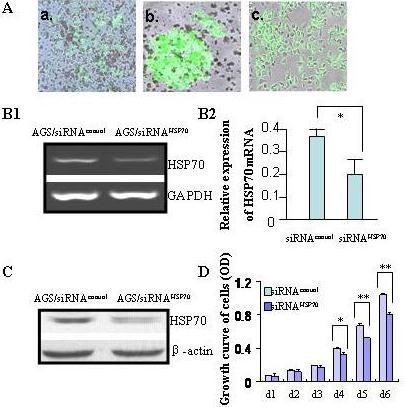
**Stable inhibition of HSP70 in AGS cell line by siRNA**. (A) AGS cells were transfected with siRNA vector targeting HSP70. a. transient transfection; b. mono-clone cell; c. stable transfection. (B) The level of HSP70 mRNA was measured by real-time PCR. Left panel showed the representative images of PCR in AGS cells transfected with siRNA/HSP70 or empty vector. Quantitative results of real-time PCR were shown in the right panel. GAPDH was used as loading control. (C) The expression profile of HSP70 in transfected AGS cells was further confirmed by immunoblotting with β-*actin *as an internal control. (D) Suppression of HSP70 by siRNA has inhibited AGS cell growth. Data are mean ± SD, *P <0.05, **P <0.01.

To further investigate the role of HSP70 depletion in *H. pylori*-associated mucosal damage, we established AGS cell lines with down-regulated HSP70 by siRNAs (Figure 2A). Suppression of HSP70 mRNA and protein in the stable transfected cell line was confirmed, which showed approximately 50% decrease in HSP70 expression as compared to vector transfected control cells (Figure 2B&2C). The reduction of HSP70 slowed down the growth of AGS cells with the morphological senescence characterized by large and membrane blebbing (Figure 2D).

### Effect of HSP70 depletion on proliferation of gastric epithelial cells with H. pylori infection

**Figure 3:**
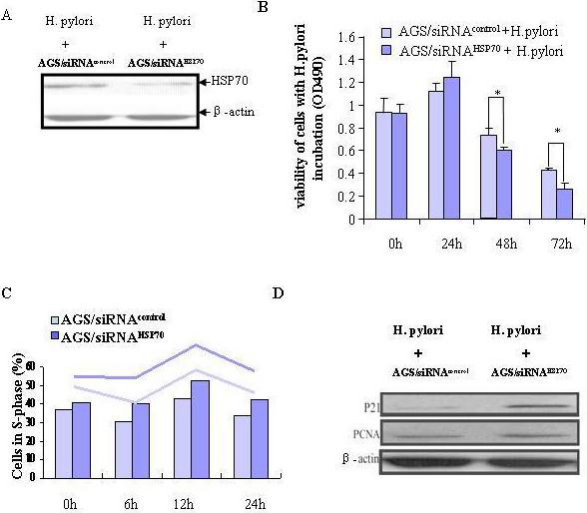
**HSP70 depletion enhanced inhibition of proliferation in AGS cells with *H. pylori *infection**. (A) Aberrant expression of HSP70 in transfected AGS cells with *H. pylori *infection was evidenced by immunoblotting. (B) HSP70 suppression further aggravated inhibitory role of *H. pylori *in cell viability in AGS cells. Data are mean ± SD, *P <0.05. (C)The effect of HSP70 depletion on proliferation was further confirmed by flow cytometre, which showed more accumulation of HSP70 siRNA transfected cells in S-phase as compared with the control vector transfected cells. (D) Protein expression of p21 and PCNA was evaluated by immunoblotting. β-*actin *was used as an internal control.

*H. pylori *inhibited cell growth accompanying suppression of HSP70 expression in AGS cells. We thus examined whether the suppressive effect of *H. pylori *on cell proliferation may result from the down-regulated HSP70. As the cells were cocultured with *H. pylori*, a much lower level of HSP70 expression was demonstrated in AGS/siRNA^HSP70 ^cells comparing with AGS/siRNA^control ^cells (Figure 3A). Viable cells were significantly reduced to 64.5% or 28.6% in AGS/siRNA^HSP70 ^cells (vs. 78.5% or 46.4% in AGS/siRNA^control ^cells, P < 0.05) following incubation with *H. pylori *for 48 h or 72 h respectively (Figure 3B).

### HSP70 depletion causes cell cycle arrest in S phase in gastric cells with H. pylori infection

To determine if HSP70 depletion mediated growth inhibition was the result of its cell cycle modulation, we investigated the effect of HSP70 inhibition on cell cycle distribution in *H. pylori*-infected AGS cells. Our results revealed a significant increase in the number of cells in the S phase in AGS/siRNA^HSP70 ^as compared with its control at different time interval (Figure 3C).

To elucidate the molecular basis by which HSP70 modulates cell cycle in gastric epithelial cell, p21 and PCNA, the well-known genes involved in the cell cycle regulation, were analyzed. Immunoblotting analysis confirmed that p21 protein but not PCNA was expressed at higher levels in the AGS/siRNA^HSP70 ^cells compared with control vector transfected cells (Figure 3D).

### HSP70 depletion induced apoptosis and pro-apoptotic proteins in gastric cells with H. pylori infection

**Figure 4:**
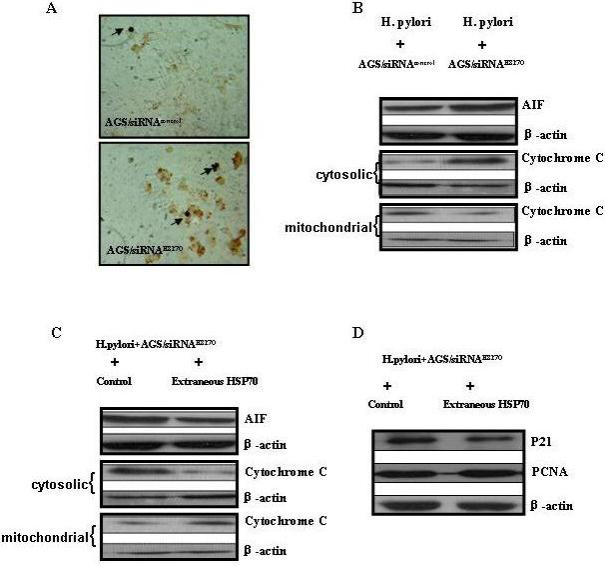
**Effects of endogenous or exogenous HSP70 on apoptosis in AGS cells with *H. pylori *infection**. (A) Representative TUNEL staining of AGS cells transfected with HSP70 siRNA or control vector following incubation with *H. pylori *for 48 hours. An increase in the number of TUNEL-positive cells (brown-stained nuclei, black arrows) was evident in HSP70 siRNA transfected cells. (B) Immunoblotting analyses were performed to determine the target pro-apoptotic proteins expression in HSP70 siRNA transfected AGS cells with *H. pylori *infection.(C)&(D) Excellular HSP70 (50 ng/ml) incorporated into the incubation medium of HSP70 siRNA transfected AGS cells and *H. pylori *(1:200) for 48 h. The function of exogenous HSP70 on the target pro-apoptotic proteins, as well as p21 and PCNA were confirmed by immunoblotting. β-*actin *was used as an internal control.

Both *H. pylori *and HSP70 have been reported to be involved in the regulation of apoptosis. To determine whether apoptosis induced in the gastric epithelium exposed to live *H. pylori *might occur due to the elimination of HSP70 expression, TUNEL staining was performed to validate the alteration of *H. pylori*-associated-apoptosis might be induced by HSP70 depletion in AGS cells. TUNEL-positive cells were much more in AGS/siRNA^HSP70 ^cells than those in the vector control cells (5.36% ± 1.22% vs 1.46% ± 0.56%, *P< 0.01; *Figure 4A).

### Identification of apoptotic genes modulated by HSP70 in AGS cells with H. pylori infection

To elucidate the molecular basis by which HSP70-suppression involved in gastric cells apoptosis, proapoptotic genes expression profile in HSP70-siRNA stably transfected AGS were analyzed by immunoblotting. The apoptosis-inducing effect by HSP70 depletion was mediated by regulating important pro-apoptotic genes including AIF and cytochrome C, as evidenced by an accumulation of AIF and the release of cytochrome c from mitochondria (Figure 4B). No considerable changes for apoptotic proteins in the down stream of the caspase-dependent apoptotic pathways, including caspase-3, caspase-6 and caspase-7 have been observed.

### Extracellular HSP70 compensated for the effect of endogenous HSP70 depletion on apoptosis and proliferation

To test whether exogenously applied HSP70 might compensate for the loss of endogenous HSP70, extracellular HSP70 (50 ng/ml) were exposed to AGS/siRNA^HSP70 ^cells with *H. pylori *infection for 48 hours. The exogenous HSP70 inhibited cytochrome c release from mitochondria to cytosol and AIF accumulation, as well as the expression of p21 (Figure 4C&4D).

## Discussion

HSP70 protects gastric mucosal cells against intrinsic and extrinsic stimuli, and maintains the proper structure and function of the gastric mucosa [14,15], suggesting induction of HSP70 might be useful for medical treatment of diseases with mucosal damage. Tsukimi Y et al. found HSP70 may facilitate the healing of acetic acid-induced gastric ulcers in rats [16]. Our previous study has demonstrated that up-regulation of HSP70 expression by Geranylgeranylacetone (GGA) interrupts the progression of atrophic gastritis in rats, as evidenced by the improvement of inflammation and glandular restoration in gastric mucosa [12]. HSP70 could protect gastric mucosa from *H. pylori*-associated gastrointestinal diseases [17]. *H. pylori *infection destroyed gastric mucosa barrier function through inducing a significant reduction of HSP70 expression in gastric epithelial cells, which was supposed to disturb gastric adaptation and facilitate H pylori to avoid host immunity [10]. We demonstrate here that suppression of HSP70 increased the sensitivity of gastric cells to *H. pylori *infection with the inhibition of cellular growth and cell cycle progression, as well as induction of apoptosis.

*H. pylori *infection damages gastric mucosa by disturbing equilibrium between apoptosis and proliferation. HSPs could reverse these inferiorities in mucosal healing. Inhibition of endogenous HSP70 slowed cellular multiplication, and enhanced the effect of *H. pylori *on cell viability. The anti-proliferation role of *H. pylori *was more evident following the further depletion of HSP70 expression. HSP70 involving in cell growth could be a cell cycle event. Rohde M et al. reported that Hela cells transfected with siRNA against HSP70 revealed an arrest in G2/M phase of cell cycle [18], which resulted in growth retardation with features of cell senescence. Our previous study have found that down-regulation of HSP70 induced S-phase arrest in AGS cells. Furthermore, we investigated the effect of HSP70 on the cell cycle of AGS cells infected with *H. pylori*, and the results showed that depletion of HSP70 induced AGS cells accumulating in S-phase independent of *H. pylori *infection. Our observation that HSP70-depletion induced S-phase arrest and p21 over-expression is in agreement with the previous report that transduction of the p21 gene resulted in S-phase arrest [19,20]. The p21 protein can inhibit DNA synthesis by interacting with PCNA [21], and plays a regulatory role in S phase DNA replication and DNA damage repair [22]. Growth arrest by p21 can promote cellular differentiation, and therefore prevents cell proliferation [23].

HSP70 depletion could make AGS more susceptible to the cytotoxicity of *H. pylori *by interference with apoptotic programs. *H. pylori *is known to cause apoptosis of gastric epithelial cells by targeting mitochondria [24,25]. Mitochondria respond to multiple death stimuli. It has been demonstrated that pro-apoptotic Bcl-2 family proteins such as Bax could induce mitochondrial membrane permeabilization and cause the release of mitochondria-mediated apoptosis signaling molecules including cytochrome c and AIF [26]. Cytochrome c triggers the caspase-dependent cascade [27], but AIF executes cell death in the absence of caspase [28-30]. *H. pylori *has been reported to trigger apoptosis in AGS cells via release of cytochrome c and AIF from mitochondria [31]. Our study demonstrated that down-regulation of HSP70 induced the further release of cytochrome c and AIF in the AGS with *H. pylori *infection, consistent with the hypothesis that HSP70 suppression could sensitize the gastric epithelial cells to the damage from *H. pylori*.

Furthermore, we evaluated the role of extracellular HSP70 in *H. pylori*-infected AGS cells, and demonstrated that extracellular HSP70 protein could partial compensate for the decreased intracellular HSP70 by reducing release of cytochrome c and AIF, which could block apoptosis in gastric cells with *H. pylori *infection. Consistently, extracellular HSP70 could also modulate the proliferation of AGS cells by inhibiting expression of p21. The exogenous HSP70 might cross the cellular plasma membrane and reduce apoptosis with the decrease of toxicity protein aggregation [32]. Exogenous HSP70 was suggested to be a trophic factor supporting cell survival [33].

## Conclusions

In conclusion, our data suggest that insufficient expression of HSP70 would render gastric epithelial cells more susceptible to *H. pylori*-induced damage than they would be if HSP70 were more abundant. The extracellular HSP70 may compensate for the deficit of endogenous HSP70 depletion. Designation to increase HSP70 protein may serve as a potential therapeutic strategy to improve the outcome of *H. pylori*-infected patients.

## Competing interests

The authors declare that they have no competing interests.

## Authors' contributions

LWL acquired the majority of data (involving assay design, cell culture, cell transfection, cell cycle and protein analysis), wrote the manuscript and contributed to the design and concept of the study. CY & LGF performed cell culture, apoptosis and viability assay. LGF performed Western blot. SLM contributed to the concept of the study and cell culture. SJM conceived the studies, oversaw the experimental work and established all the collaborations. All authors read and approved the final manuscript.

## Pre-publication history

The pre-publication history for this paper can be accessed here:

http://www.biomedcentral.com/1471-230X/11/146/prepub

## References

[B1] Choi SR Lee SA Kim YJ Ok CY Lee HJ Hahm KB Role of heat shock proteins in gastric inflammation and ulcer healing J Physiol Pharmacol 2009 60 Suppl 7 5 17 20388941

[B2] Basu S Srivastava PK Heat shock proteins: the fountainhead of innate and adaptive immune responses Cell Stress Chaperones 2000 5 443 51 10.1379/1466-1268(2000)005<0443:hsptfo>2.0.co;2 PMC312875 11189450

[B3] Ishihara T Suemasu S Asano T Tanaka KI Mizushima T Stimulation of gastric ulcer healing by heat shock protein 70 Biochem Pharmacol 2011 in press 10.1016/j.bcp.2011.06.030 21736872

[B4] Asai M Kawashima D Katagiri K Takeuchi R Tohnai G Ohtsuka K Protective effect of a molecular chaperone inducer, paeoniflorin, on the HCl- and ethanol-triggered gastric mucosal injury Life Sci 2011 88 350 7 10.1016/j.lfs.2010.12.014 21167840

[B5] Nardone G Staibano S Rocco A Mezza E D'armiento FP Insabato L Effect of helicobacter pylori infection and its eradication on cell proliferation, DNA status, and oncogene expression in patients with chronic gastritis Gut 1999 44 789 99 10.1136/gut.44.6.789 PMC1727537 10323879

[B6] Fan XG Kelleher D Fan XJ Helicobacter pylori increases proliferation of gastric epithelial cells Gut 1996 38 19 22 10.1136/gut.38.1.19 PMC1382973 8566853

[B7] Klaamas K Kurtenkov O von Mensdorff-Pouilly S Shjapnikova L Miljukhina L Brjalin V Impact of Helicobacter pylori infection on the humoral immune response to MUC1 peptide in patients with chronic gastric diseases and gastric cancer Immunol Invest 2007 36 4 371 86 10.1080/08820130601109727 17691020

[B8] Konturek JW Fischer H Konturek PC Huber V Boknik P Luess H Heat shock protein 70 (hsp70) in gastric adaptation to aspirin in Helicobacter pylori infection J Physiol Pharmacol 2001 52 153 64 11321509

[B9] Pierzchalski P Krawiec A Ptak-Belowska A Baranska A Konturek SJ Pawlik WW The mechanism of heat-shock protein 70 gene expression abolition in gastric epithelium caused by Helicobacter pylori infection Helicobacter 2006 11 2 96 104 10.1111/j.1523-5378.2006.00383.x 16579839

[B10] Huff JL Hansen LM Solnick JV Gastric transcription profile of Helicobacter pylori infection in the rhesus macaque Infect Immun 2004 72 5216 26 10.1128/IAI.72.9.5216-5226.2004 PMC517414 15322016

[B11] Axsen WS Styer CM Solnick JV Inhibition of heat shock protein expression by helicobacter pylori Microb Pathog 2009 47 231 6 10.1016/j.micpath.2009.08.002 PMC5494282 19683049

[B12] Liu WL Chen SJ Chen Y Sun LM Zhang W Zeng YM Protective effects of heat shock protein70 induced by geranyl-geranylacetone in atrophic gastritis in rats Acta Pharmacologica Sinica 2007 28 1001 6 10.1111/j.1745-7254.2007.00589.x 17588336

[B13] Gabai VL Budagova KR Sherman MY Increased expression of the major heat shock protein Hsp72 in human prostate carcinoma cells is dispensable for their viability but confers resistance to a variety of anticancer agents Oncogene 2005 24 3328 38 10.1038/sj.onc.1208495 15735699

[B14] Suemasu S Tanaka K Namba T Ishihara T Katsu T Fujimoto M A role for HSP70 in protecting against indomethacin-induced gastric lesions J Biol Chem 2009 284 19705 15 10.1074/jbc.M109.006817 PMC2740595 19439408

[B15] Rokutan K Role of heat shock proteins in gastric mucosal protection J Gastroenterol Hepatol 2000 15 Suppl 12 9 10.1046/j.1440-1746.2000.02144.x 10759215

[B16] Tsukimi Y Nakai H Itoh S Amagase K Okabe S Involvement of heat shock proteins in the healing of acetic acid-induced gastric ulcers in rats J Physiol Pharmacol 2001 52 391 406 11596858

[B17] Tomomitsu T Tomoyuki S Tomiyasu A Masakatsu N Daisuke Y Masaaki O The BB genotype of heat-shock protein (HSP) 70-2 gene is associated with gastric pre-malignant condition in *H. pylori*-infected older patients Anticancer Research 2009 29 3453 58 16-20 19661373

[B18] Rohde M Daugaard M Jensen MH Members of the heat -shock protein 70 family promote cancer cell growth by distinct mechanism Genes & Development 2005 19 570 82 10.1101/gad.305405 PMC551577 15741319

[B19] Ogryzko VV Wong P Howard BH WAF1 retards S-phase progression primarily by inhibition of cyclin-dependent kinases Mol Cell Biol 1997 17 4877 4882 10.1128/mcb.17.8.4877 PMC232340 9234744

[B20] Zhu Hongbo Zhang Lidong Wu Shuhong Teraishi Fuminori Davis John J Jacob Dietmar Induction of S-phase arrest and p21 overexpression by a small molecule 2[[3-(2,3-dichlorophenoxy)propyl] amino] ethanol in correlation with activation of ERK Oncogene 2004 23 4984 92 10.1038/sj.onc.1207645 15122344

[B21] Chen J Jackson PK Kirschner MW Dutta A Separate domains of p21 involved in the inhibition of Cdk kinase and PCNA Nature 1995 374 386 388 10.1038/374386a0 7885482

[B22] Gartel AL Radhakrishnan SK Lost in transcription p21 repression, mechanisms, and consequences Cancer Res 2005 65 10 3980 5 10.1158/0008-5472.CAN-04-3995 15899785

[B23] Abbas Tarek Dutta Anindya P21 in cancer: intricate networks and multiple activities Nat Rev Cancer 2009 9 6 400 14 10.1038/nrc2657 PMC2722839 19440234

[B24] Zhang H Fang DC Lan CH Luo YH Helicobacter pylori infection induces apoptosis in gastric cancer cells through the mitochondrial pathway J Gastroenterol Hepatol 2007 22 1051 6 10.1111/j.1440-1746.2007.04959.x 17559379

[B25] Chiozzi V Mazzini G Oldani A Sciullo A Ventura U Romano M Relationship between Vac A toxin and ammonia in Helicobacter pylori-induced apoptosis in human gastric epithelial cells J Physiol Pharmacol 2009 60 23 30 19826178

[B26] Yang J Liu XS Bhalla K Kim CN Ibrada AM Cai JY Prevention of apoptosis by Bcl-2:release of cytochrome c from mitochondria blocked Science 1997 275 1129 32 10.1126/science.275.5303.1129 9027314

[B27] Garland JM Rudin C Cytochrome c Induces Caspase-Dependent Apoptosis in Intact Hematopoietic cells and overrides apoptosis suppression mediated by bcl-2, growth factor signaling, MAP-Kinase-Kinase, and malignant change Blood 1998 92 1235 46 9694712

[B28] Joza N Susin SA Daugas E Stanford WL Cho SK Li CY Essential role of the mitochondrial apoptosis-inducing factor in programmed cell death Nature 2001 410 549 54 10.1038/35069004 11279485

[B29] Susjin SA Daugas E Ravagnan L Samejima K Zamzami N Loeffler M Two distinct pathways leading to nuclear apoptosis J Exp Med 2000 192 571 80 10.1084/jem.192.4.571 PMC2193229 10952727

[B30] Cregan SP Dawson VL Slack RS Role of AIF in caspase-dependent and caspase-independent cell death Oncogene 2004 23 2785 96 10.1038/sj.onc.1207517 15077142

[B31] Ashkorab H Dashwood RH Dashwood MM Zaidi SI Hewitt SM Green WR *H. pylori*-induced apoptosis in human gastric cancer cells mediated via the release of apoptosis-inducing factor from mitochondria Helicobacter 2008 13 506 17 10.1111/j.1523-5378.2008.00646.x PMC7322629 19166416

[B32] Novoselova TV Margulis BA Novoselov SS Treatment with extracellular HSP70/HSC70 protein polyglutamine toxicity and aggregation J Neurochem 2005 94 597 606 10.1111/j.1471-4159.2005.03119.x 15992387

[B33] Robinson MB Tidwell JL Gould T Extracellular heat shock protein 70: a critical component for motoneuron survival J Neurosci 2005 25 9735 45 10.1523/JNEUROSCI.1912-05.2005 PMC6725726 16237177

